# Review of the accreditation of digital forensics in China

**DOI:** 10.1080/20961790.2018.1503526

**Published:** 2018-10-04

**Authors:** Hong Guo, Junlei Hou

**Affiliations:** a Shanghai Key Laboratory Scienceof Forensic Medicine, Shanghai Forensic Service Platform, Academy of Forensic Science, Shanghai, China;; b Cyber Security Department, Ministry of PRC, Beijing, China

**Keywords:** Accreditation, digital forensics, forensic science, methodology, personnel training

## Abstract

As a result of the many developments in information technology, digital evidence plays an increasingly important role in criminal and civil litigation. Because digital evidence is necessary for litigation, the judicial system must be assured of its accuracy, reliability, and verifiability, which can be assured by accreditation. This paper focuses on a comparison of the evolution of the accreditation of digital forensics internationally and domestically, discusses the existing problems that such accreditation encounters, and proposes the corresponding solutions. Moreover, this paper discusses the future of digital forensic laboratory accreditation and its implementation.

## Introduction

Because of the many developments in information technology, digital evidence plays an increasingly important role in criminal and civil litigation. Today, digital evidence is now used to prosecute all types of crimes, not just cybercrime. Because many types of digital evidence may be necessary for litigation, the judicial system has to be assured of its accuracy, reliability, and verifiability. Correspondingly, establishing the chain of custody when authenticating digital evidence in the courtroom is extremely important and absolutely necessary. The chain of custody must account for the seizure, storage, transfer, and condition of the evidence. This goes far beyond just finding and extracting the data, examining and interpreting its relevance, and generating a report.

Digital evidence can be active, deleted, hidden, encrypted, or overwritten, and cannot be seen by the naked eye. When dealing with digital evidence, relevant scientific principles relating to the collection, processing, and examination of evidence must be followed. Digital forensics is the “process of identifying, preserving, analysing, and presenting digital evidence in a manner that is legally acceptable in any legal proceedings” [1]. It is currently one of the fastest growing sections in the crime lab, and its importance to law enforcement is growing exponentially.

In China, the need to ensure the acceptance and admissibility of digital evidence is increasing: the quality of the appraisal directly determines the social credibility of the result and further affects the ability of judicial appraisal to achieve its purpose of serving litigation and promoting judicial fairness. At present, accreditation has become a common practice in institutional management appraisal in forensic science.

Accreditation is an internationally recognized assessment method that provides a standard to ensure that the digital evidence obtained from the examination is accurate, technically competent, and valid according to accepted quality assurance practices. It increases public confidence and trust in the evidence used within the criminal justice system. Therefore, accreditation is an important means for China's forensic laboratories to become standardized, scientific, and internationally recognized.

This paper compares the evolution international and domestic digital forensics accreditation. Further, it discusses the existing problems that the accreditation of digital forensics encounters and proposes the corresponding solutions. The conclusion of this paper discusses the future of accreditation of digital forensic laboratory and its implementation.

## Evolution of international digital forensics accreditation

This section reviews the development of international digital forensics accreditation. The accreditation of digital forensic laboratories started late but developed rapidly. In 2002, International Laboratory Accreditation Cooperation (ILAC) issued ILAC G-19 “Guidelines for Forensic Science Laboratories”, to “… provide guidance for laboratories involved in forensic analysis and examination by providing application of ISO/IEC 17025”. ILAC G19 (2002) explicitly includes computer analysis as part of the forensic science laboratory accreditation. In 2014, Working Group 10 of the ILAC Accreditation Committee (ILAC AIC WG10) issued ILAC G19 “Modules in a Forensic Science Process”, which combines the areas of the International Organization for Standardization/International Electrotechnical Commission (ISO/IEC) 17020 and ISO/IEC 17025. ILAC G19 maintains the scope of computer analysis, dividing it into three sub-scopes: computers (hardware and software), recovery of information from electronic devices and media, and mobile computerized devices (including phones, Global Positioning System (GPS) devices, and personal digital assistants) [2].

### International digital forensics accreditation

Begun in 1982, the American Society of Crime Laboratory Directors/Laboratory Accreditation Board (ASCLD/LAB) is the largest provider of accreditation services to public crime laboratories. The digital evidence discipline was accredited by ASCLD/LAB in 2003 and divided into four subdisciplines: computer forensics, audio analysis, video analysis, and imaging analysis. In 2005, the name of this discipline was changed to digital and multimedia evidence. The ASCLD/LAB used ISO/IEC 17025 for its accreditation requirements [3]. Then, in April 2016, the ASCLD/LAB was acquired by and merged into the American National Standards Institute-American Society for Quality (ANSI-ASQ) National Accreditation Board (ANAB). The ANAB uses both ISO/IEC 17025 and 17020 for its accreditation requirements, and the subdisciplines were merged into one discipline: digital evidence. Presently, 72 forensic inspection and forensic laboratories are accredited by the ANAB.

In England and Wales, the Forensic Science Regulator's (FSR) Code of Practice stipulates that all providers of digital forensic services to the criminal justice system must be accredited to ISO/IEC 17025 for forensic imaging, data extraction, and analysis of data on computer and mobile devices. Data capture and analysis from networks are set to become mandatory from October 2018. Other areas that are being considered for mandatory accreditation include cell site analysis and communications data, as well as the capture and analysis of social media and open source data [4]. As of October 2017, 12 legal entities had been granted accreditation for imaging conventional hard drives, solid state devices, and peripheries; three legal entities had been granted accreditation for the data extraction and analysis of the same types of drives; six legal entities had been granted accreditation for the logical and physical capture, analysis, and processing of data from mobile phones; and two legal entities had been granted accreditation for the processing and enhancement of closed-circuit television (CCTV) [5].

In Australia, the forensic science community, through the Australia New Zealand Policing Advisory Agency (ANZPAA), the National Institute of Forensic Science (NIFS), and the Senior Managers of Australia New Zealand Forensic Laboratories (SMANZFL), partnered with the National Association of Testing Authorities (NATA) to develop a forensic science accreditation program. The primary international standard for the NATA program is ISO/IEC 17025. In November 2008, NATA added electronic evidence to its forensic science accreditation program. NATA included just two subdivisions: data preservation and data analysis [6]. Today, like the ANAB, NATA has changed its discipline to digital evidence.

### Standards for international digital forensics accreditation

A 2016 report by Athanas notes that ILAC currently has 87 recognized accreditation bodies and 51 organizations accredited to perform digital forensics, of which 48 are accredited under ISO/IEC 17025 and two are accredited under ISO/IEC 17020 [7].

According to Athanas, ISO/IEC 17025 is the most widely recognized accreditation of digital forensics. ISO/IEC 17025 is an international quality standard used by accreditation bodies worldwide for the accreditation of both testing and calibration laboratories and includes management and technical requirements. ISO/IEC 17025 is derived from ISO/IEC Guide 25 “General Requirements for the Competence of Testing Laboratories”, which issued three versions: ISO/IEC 17025:1999, ISO/IEC 17025:2005, and ISO/IEC 17025:2017. ISO/IEC 17025 has proven to be useful for quality and basic competency management in digital forensics. The ILAC notes the following reasons for using accreditation based on ISO/IEC 17025: recognition of testing competence, performance benchmarking, marketing advantage, and international laboratory recognition [8].

However, there has been discussion about whether ISO/IEC 17025 is right for digital forensics since the standard became mandatory in the United Kingdom for all digital forensic laboratories. Many digital forensic examiners are opposed ISO/IEC 17025 for many reasons including the costs, lack of understanding, poor implementation, and impact of inconsistency [9].

In addition, there is a recent effort to introduce ISO/IEC 27037:2012 into the discipline of digital evidence. ISO/IEC 27037:2012 defines digital evidence and describes its three main governing principles: relevance, reliability, and sufficiency. The initial digital evidence-handling processes (identification, collection, acquisition, and preservation) are also detailed through descriptions of key components within the process. We hence believe that ISO/IEC 27037:2012 is the best fit for digital forensic laboratories.

### Proficiency testing for international digital forensics

A proficiency test is an analytical test used to evaluate the technical competence of examiners, technical support personnel, and the overall quality of a forensic science service provider. For forensic science service providers, proficiency testing helps ensure that quality work is being delivered and maintained, therefore, it is an effective quality assurance tool.

In recent years, proficiency testing activities in the area of computer forensics have also developed rapidly. For example, Collaborative Testing Services (CTS) interlaboratory testing programs are recognized by several organizations in the world, including the United States, the United Kingdom, France, the Netherlands, Canada, and Japan. In 2015, 52 institutions participated in the CTS Forensics Testing Program in the field of Digital and Multimedia Evidence and 32 returned results. In 2016, 88 institutions participated and 80 returned results, and in 2017, 105 institutions participated and 80 returned results.

## Evolution of digital forensics accreditation in China

The accreditation of forensic institutions in China has been promoted because of laws and regulations. Prior to 2005, the accreditation of forensic science was fully voluntary. As a result, the number of accredited forensic institutions was very small. With the reform of the judicial system, to improve the credibility of the judiciary and regulate judicial appraisal work, the “Decision of the Standing Committee of the National People's Congress on issues of administration of judicial authentication”, referred to as the 2.28 Decision, was passed at the 10th Meeting of the Standing Committee of the Fourteenth National People's Congress on 28 February 2005, and was put into practice on 1 October 2005. It clearly stipulates that “the laboratories providing forensic services must be a measurement certificated laboratory or an accredited laboratory”. The issuance of the 2.28 Decision has become an important milestone in the implementation of certification and accreditation for forensic science institutions.

On 29 September 2005, the Ministry of Justice of the People's Republic of China issued the “Administrative Regulation on the Registration of Judicial Forensic Institutions”, on 7 November 2005, the Ministry of Public Security of the People's Republic of China issued the “Administrative Regulation on the Registration of Judicial Forensic Institutions of Public Security”, and on 30 November 2006, the Supreme People's Procuratorate of the People's Republic of China issued the “Administrative Regulation on the Registration of Judicial Forensic Institutions of the Supreme People's Procuratorate”. These three documents all require that laboratories providing forensic services must be a measurement certificated laboratory or an accredited laboratory. The issuance of the above regulations has transformed the certification and accreditation of China's forensic institutions from a voluntary act to a mandatory one, which makes certification and accreditation two basic conditions for the forensic institutions. As a result, certification and accreditation become important evaluation methods for quality management in the forensics field.

### Digital forensics accreditation in China

According to the 2.28 Decision, there are two main organizations that are currently licensed to provide digital forensic service in China: judicial forensic institutions registered by provincial judicial administrations and judicial forensic institutions of public security registered by the provincial public security organs. Judicial forensic institutions of the procuratorate and forensic institutions of the emergency management department are generally incorporated into the administration of forensic institutions, and institutions of the customs anti-smuggling system are incorporated into the administration of the public security organs.

Compared with developed countries, the accreditation of China's digital forensics started later, but it developed rapidly. By the end of April 2018, more than 100 organizations providing digital forensic services had been accredited, most of which are judicial forensic institutions of public security. At present, these accredited organizations have vigorously promoted the standardization of their work, improved their abilities and quality management, and effectively achieved their aim of combating cybercrime.

### Standards for digital forensics accreditation in China

As to the standards for accreditation, accredited digital forensic organizations initially used either ISO/IEC 17025 or ISO/IEC 17020. To determine appropriate standards for accreditation, on 26 August 2013, the China National Accreditation Service for Conformity Assessment (CNAS) issued CNAS-CL08:2013 “Accreditation Criteria for the Competence of Forensic Units”, which is in accordance with ISO/IEC 17025:2005 and adopts part of the ISO/IEC 17020:2012 and ILAC G19, as a reference for judicial authentication and forensic science for accreditation. CNAS-CL08 was revised as CNAS-CL08:2018 on 1 March 2018 to accommodate the changes in ISO/IEC 17025:2017.

On 5 December 2013, CNAS released CNAS-AL13 “Discipline of Accreditation for Forensic Units”, and the revised version was issued on 1 June 2015. The Digital Evidence discipline was divided into three subdisciplines: Data Extraction, Preservation and Recovery, Authentication of Electronic Data, and Consistency and Similarity of Electronic Data. Detailed information about the Digital Evidence discipline is shown in Table 1 [10].

**Table 1.** Discipline of accreditation for forensic units.

Discipline	Subdisciplines and devises
Digital Forensics	2401 Data Extraction, Preservation and Recovery
	01 Computer Storage media
	02 Embedded Systems
	03 Mobile Devices (including mobile phones)
	04 Smart Cards and Magnetic Cards
	05 Digital Devices
	06 Network Data (including Internet data)
	07 Computer System Live Data (specifically running system data extraction)
	2402 Authentication of Electronic Data
	01 Electronic Signatures
	02 E-mail
	03 Instant Messaging
	04 Electronic Documents
	05 Database
	2403 Consistency and Similarity of Electronic Data
	01 Software
	02 Digital Documents
	03 Integrated Circuit (including chips)

On 1 April 2014, according to the characteristics and requirements of digital forensics, CNAS issued CNAS-CL272010 “Guidance on the Application of Laboratory Accreditation Criteria in the Field of Electronics”. On 1 April 2014, CNAS revised the guidance and issued CNAS-CL27:2014 “Guidance on the Application of Accreditation Criteria for the Competence of Forensic Units in the Field of Digital Forensics”, On 18 April 2018, CNAS revised the guidance again and issued CNAS-CL08-A001:2018 “Guidance on the Application of Accreditation Criteria for the Competence of Forensic Units in the Field of Digital Forensics”.

### Proficiency testing for digital forensics in China

In China, proficiency testing is an important means of assessing and supervising the technical capabilities of laboratories. The results of the proficiency testing can also provide important references for supervisory authorities. According to CNAS-AL07:2015 “Proficiency Testing Area and Frequency”, accredited digital forensic laboratories must participate in proficiency testing at least once every 2 years in the field of digital evidence [11].

At present, there are two proficiency testing providers in the field of digital forensics in China: the Academy of Forensic Science and Shanghai Stars Digital Forensic Centre. The digital forensics proficiency testing activities organized by the Academy of Forensic Science have been held annually since 2009. The test participants are mainly forensic institutions and most disciplines in the field of digital forensics are covered. In 2017, more than 100 laboratories participated. The proficiency testing activity organized by the Shanghai Stars Digital Forensic Centre has been jointly organized with the Cyber Security Bureau in the Ministry of Public Security since 2011. Therefore, the participants are mainly judicial forensic institutions of the Cyber Security Bureau. It only tests proficiency in the discipline of Data Extraction, Preservation, and Recovery. This test is also annual, and, in 2016, nearly 500 laboratories participated.

In addition, since 2012, the Criminal Investigation Bureau of the Ministry of Public Security has jointly held a proficiency testing program in conjunction with CNAS, which also includes the digital forensic program. The participating organizations are limited to public security forensics laboratories.

## Challenges and solutions for digital forensics accreditation

Although China has made rapid progress with respect to the technology, standards, and products in the field of digital forensics, as well as digital forensics accreditation work, there are still many problems and challenges in this field because of weak foundations and a lack of awareness.

### Training

Because any digital evidence presented should meet the requirements regarding the acceptance and admissibility of digital evidence, judicial forensic institutions should standardize various aspects of forensic work such as personnel, equipment, facilities and environment, management, methodology, security, and procedures. Personnel is the most important part of the overall quality management system. Therefore, digital forensics has extremely high requirements for experience and professionalism that are clearly stated in CNAS-CL08 “Administrative Regulations on the Registration of Judicial Forensic Staff”, and CNAS-CL27 (CNAS-CL08-A001) “Administrative Regulations on the Registration of Forensic Staff of Public Security Organs”.

However, professionalism is not innate; maintaining and increasing professionalism and ensuring quality requires scientific training and systematic study. At present, China's training in the field of digital forensics is far behind international training and certification. Although related courses have been set up in public security as well as political and law universities, the coursework is far from actual investigation and the results are not satisfactory. Most digital forensic institutions can only operate by participating in the proficiency testing organized by CNAS and proficiency testing (PT) provider.

At present, there is no authoritative training system and certification for digital forensics. First, there is a lack of training types, including not only different training plans for people with different backgrounds and knowledge levels but also training plans for different areas of electronic data and different levels of difficulty. Second, there is a lack of authoritative textbooks. Currently, there are no systematic teaching materials on the market for digital forensics because of the lack of training certification design at the top level. Third, there are no experienced teachers; some colleges and university teachers have solid basic theoretical knowledge but lack practical work experience in the field. Field-based forensics investigators also lack the opportunity to present themselves as teachers and turn their own experience into a basis for teaching. Fourth, there is a lack of authoritative training. According to the relevant regulations, a person can obtain forensic certification as long as he or she has reached a certain level of relevant knowledge and years of relevant work. However, for digital forensics, this is far from sufficient, and it is necessary for judicial forensic departments to formulate a set of effective certifications as soon as possible.

Internationally, training in the area of digital forensics has become a mature industry. There are both product-based certification trainings such as EnCase Certified Examiner (EnCE) and AccessData Certified Examiner (ACE). In addition, there are certifications based on entire digital forensic technology systems such as the International Association of Investigative Specialists (IACIS) Certified Forensic Computer Examiner (CFCE) certification, International Society of Forensic Computer Examiners Certified Computer Examiner (CCE) certification, SANS Global Information Assurance Certification (GIAC) Certified Forensic Examiner (GCFE), GIAC Certified Forensic Analyst (GCFA), and EC-Council Certified Hacking Forensic Investigator (CHFI).

To achieve digital forensics training within China, it is first necessary to establish a digital forensics training system that suits China's national conditions. There must be a top-level design conducted by national authorities. At the same time, it is necessary to learn from international training experience, absorb advanced concepts from abroad, establish professional training lessons, faculty teams, and a certification system with Chinese characteristics. At present, the corresponding agencies of the Ministry of Public Security and the Ministry of Justice have started compiling a series of textbooks on digital forensics, and many police colleges are also starting to offer associate's, bachelor's, and master's degrees in the area of digital forensics. In addition, the Ministry of Public Security and the Ministry of Justice have strengthened their training through annual proficiency testing.

### Methodology

Because electronic data does not belong to statutory evidence, the standardization of electronic evidence in China has stagnated for a long time. However, in recent years, with the emphasis on related standards work, the standardization of digital forensics has developed rapidly. At present, four national standards (Guojia Biaozhun in Chinese, abbreviated as GB), 19 public safety industry standards (Gonggong Anquan in Chinese, abbreviated as GA), and 10 forensic technical specifications (Sifa Jianding in Chinese, abbreviated as SFJD), have been issued, as shown in Table 2.

**Table 2.** Digital forensic standards in China.

Category	Standard No.	Standard name
National standard	GB/T 29360-2012	Technical specification for electronic forensics data recovery
	GB/T 29361-2012	Technical specification for electronic forensics file identification
	GB/T 29362-2012	Technical specification for electronic forensics data search
	GB/T 31500-2015	Information security technology – requirements of data recovery services for storage media
Public safety industry standard	GA/T 754-2008	Requirements and test methods for electronic data storage duplication tools
	GA/T 755-2008	Requirements and test methods for write blockers for electronic data storage
	GA/T 756-2008	Discovery, extraction, and preservation methods for evidence data on digital devices
	GA/T 757-2008	Test methods for program functions
	GA/T 828-2009	Software function test technical specifications for electronic forensics
	GA/T 829-2009	Software identification technical specifications for electronic forensics
	GA/T 976-2012	General method for electronic data identification of forensics
	GA/T 977-2012	Electronic signatures of forensics and identification documents
	GA/T 978-2012	Technical verification methods for unauthorized online games
	GA/T 1069-2013	Technical specifications for electronic forensics mobile phone examination
	GA/T 1070-2013	Technical specifications for forensic computer switch time examination
	GA/T 1071-2013	Technical specifications for electronic forensics Windows operating system log examination
	GA/T 1170-2014	Examination methods for evidence collection from mobile terminals
	GA/T 1171-2014	Examination methods for similarity comparison of integrated circuit chips
	GA/T 1172-2014	Technical methods for e-mail examination
	GA/T 1173-2014	Technical methods for examination of instant messaging data
	GA/T 1174-2014	General methods for capture of live electronic evidence data
	GA/T 1175-2014	Technical methods for examination of software similarity
	GA/T 1176-2014	Technical methods for examination of web browser history data
Forensic technical specification	SF/Z JD0400001-2014	Specification for electronic forensics data identification
	SF/Z JD0401001-2014	Specification for electronic data storage duplication tool
	SF/Z JD0403001-2014	Specification for examination of software similarity
	SF/Z JD0402001-2014	Specification for e-mail examination
	SF/Z JD0400002-2015	Specification for capture of live electronic evidence data
	SF/Z JD0402003-2015	Specification for examination of instant messaging
	SF/Z JD0403003-2015	Specification for computer system user operation behaviour inspection
	SF/Z JD0403002-2015	Specification for destructive program inspection
	SF/Z JD0401002-2015	Specification for mobile electronic data extraction operation
	SF/Z JD0402002-2015	Specification for database data authenticity

Compared with international standards, the standards and specifications in the field of digital forensics issued in China appear to be numerous, but due to the lack of coordination and unified planning among the Organization for Standardization, the duplication of standard content is a serious issue. Taking the GB standard as an example, of the four GB standards, two repeat content because they are from different departments. Not only are GA standards and SFJD specifications almost completely duplicated, but GA standards themselves are also subject to duplication.

One of the basic requirements of a standard is that it must be unified, and it must be recognized by all departments and communities. The phenomenon of standard repetition means that many digital forensic laboratories often fail to determine which standard should be used when applying for accreditation. Even many standards that have been selected by accredited electronic data authentication laboratories have been erroneous.

In addition, the standards drafters only pay attention to the number of declarations and do not pay attention to the quality, leading to serious mistakes in certain standards. Digital forensic laboratories will inevitably incur risks if they operate in accordance with such standards in actual cases. Once standards are established, they will not be revised for some time. Taking the industry standard as an example, a standard from project to release has a cycle of about three years. To carry out a revision generally takes more than five years. This is just the cycle of establishment, and it is not yet the actual release cycle.

**Figure 1.**
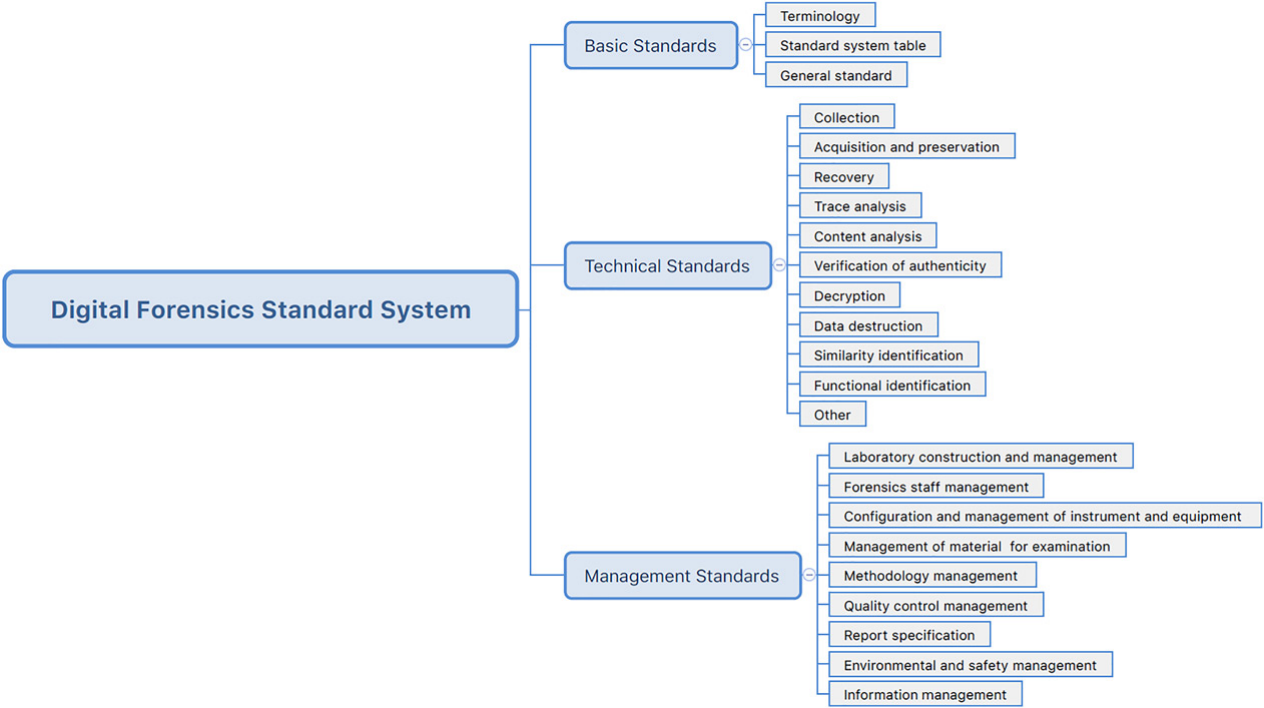
Digital forensics standard system proposed by the Ministry of Justice (top three levels).

In view of the current confusions in the standards, China has begun to establish a standard system for digital forensics. At present, referring to international research achievements and standards, the National Technical Committee for Criminal Technology Standardization Technical Committee, considering China's actual national characteristics, has embarked on the development of an electronic data forensic standard system and related standards that meet China's actual work requirements. The draft of the standard system has been reviewed by industry experts. In addition, the Ministry of Justice also started drafting of a standard system for the forensic authentication of electronic data, which is shown in Figure 1.

### Equipment and software

As technology continues to rapidly change and evolve, so has the complexity of the forensic analysis of digital evidence, and hence new forensic techniques and tools are necessary for digital forensic investigators. Without appropriate forensic tools, the efficiency of the forensic process and the accuracy of forensic results will be substantially reduced. Therefore, the success of digital forensics depends to a large extent on whether the forensic personnel are skilled and have sufficient, reliable, and efficient forensic tools. CNAS-CL08 also clearly stipulates that “the equipment and its software used for forensic examinations shall be capable of achieving the accuracy required and shall comply with specifications relevant to the tests concerned”.

At present, China's product development ability in the field of digital forensics is at the forefront globally. However, the ability to test forensic products is at a significant disadvantage compared with developed countries. Neither the test standards for forensic tools nor the standardized test images for matching have been established. Many forensic products are often offered commercially without careful testing to seize the market. Especially in mobile phone forensics products, different versions of the same product often produce different results, which seriously affect the credibility of the forensic results.

At present, China has not yet established an access system for forensic equipment. The only testing standards for forensic tools are GA/T 754-2008 and GA/T 755-2008. With the continuous development of forensic technology, a large number of new types of evidence have appeared. Mobility, encryption, cloud storage, the Internet of things, and blockchain have dramatically changed the landscape of digital forensics. The emergence of tools is not sufficient for combating actual crime. China should actively study the testing mechanisms of the forensic equipment established by the National Institute of Standards and Technology and establish test standards for forensic tools that suit the actual conditions of our country and meet the needs of the work. The products should be tested by authorities to ensure the validity, reliability, and stability of digital forensics. Fortunately, the issue forensic tool reliability has attracted widespread attention. How to evaluate such tools has also been put on the agenda of the relevant agencies.

## Conclusion

With the continuous advancement of the trial-centred criminal litigation system, the Supreme People's Court, the Supreme People's Procuratorate, and the Ministry of Public Security jointly issued the “Regulations on Several Issues Concerning the Collection, Examination and Judgment of Electronic Data in Handling Criminal Cases” in 2016 to further regulate electronic evidence collection and review judgments. As the status of the accreditation work in the field of digital forensics in China continues to increase, it has received increasingly more attention, which has also played a positive role in promoting the standardization of evidence collection procedures and the realization of judicial justice.

It can be foreseen that, under the correct leadership of CNAS, domestic digital forensic institutions will continue to improve their management and technical capabilities of the organization through accreditation activities, so as to improve the admissibility of evidence, and realize judicial justice.

## Compliance with ethical standards

This article does not contain any studies with human participants or animals performed by any of the authors.
